# The Institutional Worker

**Published:** 1921-02-05

**Authors:** 


### THE INSTITUTIONAL WORKER.

*Being a Special Supplement to "The Hospital."*

### INSTITUTION FACTS AND FIGURES.

### The Question Box.

### CHECKS ON WASTE.

#### Answer to January Question.

The question was:

**Describe methods for keeping a check on the use of drugs and dressings in the hospital wards and operating-theatre with a view to prevent waste.**

**"Vigoral" replies:—**

Now that the cost of drugs and surgical dressings is so high, as compared with pre-war prices, it is absolutely necessary that an endeavour should be made to eliminate all waste.

Drugs.

These should not be supplied from the dispensary without a written order—in the case of prescriptions for medicines and special preparations the bed-card will usually constitute the order, whilst "stock" preparations are generally ordered in a "stock" book signed by the sister-in-charge or resident medical officer. The hospital pharmacist should, when unusual quantities of stock medicines are ordered, verify the quantities from the bed-cards ordering the medicines. A very useful plan is also for a responsible official to overhaul the cupboard of "stocks" and return preparations not required, along with any surplus, to the dispensary.

Many special preparations, tablets, pills, etc., thus returned can be utilised for other patients as required, the dispensary acting as a kind of clearing-house in the matter. In the experience of the writer, waste is most frequently found when dealing with such articles as methylated spirit, turpentine, dusting powder, ointments, etc. Methylated spirit and turpentine (rectified) should never be allowed for cleaning up sinks, wash-basins, etc.—a little dry soap will do just as well. Again, dusting powder is frequently used much too lavishly, as also are zinc and other ointments. In the operating theatre methylated spirit used for instruments should be poured off into a bottle and used again, or for other purposes in the wards.

Stoppers on bottles of very volatile preparations like æther, chloroform, collodion, etc., should be securely fastened when not in use, for if not the whole of the bottle contents may evaporate through a missing stopper.

Oxygen and nitrous oxide, though perhaps hardly to be regarded as "drugs," are often handled carelessly, with consequent loss of the cylinder contents: the valve should never be opened "full," but the key turned to allow a *gentle* flow of the gas.

Surgical Dressings.

The best method to check waste in the use of surgical dressings is to have a "requirement card" or book with all usual articles tabulated, columns for thirteen weeks and a "total" column. Dressings from dispensary or stores should be issued on a fixed day and time, once a week. A special "summary card" should be kept of the consumption of each article for the quarter in every ward. Surgical wards will generally average varying figures, and regard must be had for any special cases requiring large quantities of dressings in making comparison between the wards. A good incentive to economical use is to provide the ward sister with a copy of the quarterly consumption summaries for a period, say, of two years.

Now that cotton wool is so expensive, a useful substitute in such cases as colotomy is cellulose wadding, also dried moss; the use of large quantities of absorbent wool ought to be regarded as extravagance in these cases. Boric lint, too, is frequently used in pieces much larger than is necessary. Bandages can be used over again, and each ward should have a bandage roller for the purpose. Hospital strapping is now almost prohibitive in price, and should not be allowed for use in labelling bottles, tins, etc. Lint even now is about three times the pre-war price, and probationers and nurses should be informed that its use must be rigidly restricted to definite surgical uses.

The writer had occasion to inquire as to the abnormal consumption of hospital lint in the babies' ward at a hospital, and found that it was used for infants' napkins, which, when soiled, were thrown away! Again, in some institutions the practice of using lint as polishing cloths is not unknown! All such cases require careful watching by the sister who is responsible for the charge of ward stores. Finally, I would suggest that the matron or her deputy should make a regular inspection of the soiled-dressings bins to ascertain any excessive use of absorbent wool, bandages, etc., and every member of the nursing staff should be encouraged to express her ideas on the prevention of waste.

### Question for February.

### HYGIENE INSTRUCTION AT WELFARE CENTRES.

**Outline a scheme for the collective instruction in hygiene of expectant and nursing mothers, and also of voluntary and salaried workers, at a Maternity and Child Welfare Centre. The expenses of such instruction being provided for by the grant offered for this purpose under the Maternity and Child Welfare Act 1918.**

**(A minimum payment of 10s. 6d. will be made for each published answer.)**

RULES.

The following rules must be observed:—

1. Contributions must be written on one side of the paper. Conciseness and terseness are desirable features. The MS. must bear the name and address of the sender and be accompanied by coupon to be cut from the back cover (inside page) of the current issue of The Hospital. A pseudonym must be chosen if the name is not to be published.

2. Contributions must be addressed to the Editor of The Hospital Institutional Worker Supplement, 28 & 29 Southampton Street, Strand, London, W.C. 2, should reach him before the end of the current month, and be marked in the left-hand corner "Facts and Figures."

### Matrons' and Sisters' Appointments.

KNIGHTSWOOD HOSPITAL (SANATORIUM), GLASGOW.—Miss Gertrude E. Newton has been appointed sister. She was trained at the Essex County Hospital, Colchester, and at the City Hospital, Edinburgh. She is a member of the College of Nursing.

SWANSEA UNION INFIRMARY.—Miss Agnes Harvey has been appointed sister. She was trained at Barnsley Infirmary.

EDINBURGH CITY HOSPITAL.—Miss Annie Rodgers has been appointed sister in the sanatorium wards. She was trained in general nursing at the Royal Infirmary, Edinburgh, and in fever training at the City Hospital, Edinburgh, and during the war saw service in the East.

ROYAL CORNWALL INFIRMARY, TRURO.—Miss Olga M. Frost has been appointed sister. She was trained at this hospital, and later was theatre staff nurse at the Hospital for Women, Soho Square, and charge nurse at the Frodingham Accident Hospital.

BOROUGH HOSPITAL, MERTHYR TYDFIL.—Miss Ruby A. Kidd has been appointed sister. She was trained at Dr. Steevens' Hospital, Dublin, at the Dublin Children's Orthopædic Hospital, and at the Clonskea Fever Hospital, Dublin. During the war she served in France and in various war hospitals in England under the Q.A.I.M.N.S.R.

SOLIHULL UNION INFIRMARY.—Mrs. Alice Bryan has been appointed superintendent nurse. She was trained at University College Hospital, and has been superintendent nurse at the Guardians' Institution, Bury St. Edmunds.

THROAT HOSPITAL, GOLDEN SQUARE.—Miss E. E. Simpson has been appointed night sister. She was trained at the Royal Surrey County Hospital, Guildford, and has subsequently held a sister's post at Bromley Cottage Hospital, St. Mark's Hospital, City Road, and at the National Hospital, Queen Square.

VICTORIA HOSPITAL, BLACKPOOL.—Miss Agnes Wiggins has been appointed out-patient sister. She was trained at the Royal Southern Hospital, Liverpool, where she was subsequently for one year on the private staff. Later she joined the staff of the Children's Hospital, Leasowe. Miss Wiggins holds the massage certificate of the National Hospital and University College Hospital.

### Annual Prize-giving, Bath Union Infirmary.

AT the meeting of the Board of Guardians on January 19 the Chairman presented the gold medal to Nurse Winifred Elsie Ponter, who was described by Dr. Duckett, the medical officer, as the best probationer they had had during the seven years' training of nurses in Bath. The silver medal was gained by Nurse Lawrie and the bronze medal by Nurse Wood. High appreciation of the work of the superintendent nurse, Miss Turner, was expressed. The lack of operative surgery is proving a great bar to the development of the training-school, and the Guardians are threatened with the prospect of losing recognition from the Ministry of Health unless they bring the infirmary up to date.

### Missionary Working Parties.

SINCE the war taught everyone to make hospital necessaries, the Zenana Bible and Medical Mission, as well as the C.M.S. Medical Mission Branch, has enlisted the services of numerous helpers to prepare bandages, muslin swabs, operating overalls, aprons, and a host of other requirements for mission hospitals Two all-day working parties will be held at 33 Surrey Street, Strand, on February 14 and 28, from 10.30 A.M. to 5 P.M., with dinner interval, and nurses who have a day to spare will be specially welcomed by the convener, Mrs. Sydney Edwards.

